# Intercropping and *Rhizobium* Inoculation Affected Microclimate and Performance of Common Bean (*Phaseolus vulgaris L*.) Varieties

**DOI:** 10.1155/2022/3471912

**Published:** 2022-12-06

**Authors:** Shemeles Tesfaye Shumet, Tewodros Ayalew, Amsalu Gobena Roro, Hussien Mohammed Beshir

**Affiliations:** ^1^Department of Crop Sciences, Afar Pastoral and Agro Pastoral Research Institute, P.O. Box 16, Semara, Ethiopia; ^2^School of Plant and Horticultural Sciences, Hawassa University, College of Agriculture, P.O. Box 05, Hawassa, Ethiopia

## Abstract

A field experiment was carried out at Hawassa, during the 2020 cropping season with the objective to evaluate the impact of maize-common bean intercropping and Rhizobium inoculation on microclimate, growth, and yield of common bean varieties. Treatments consisting of two common bean varieties, two levels of inoculation and three spatial arrangements of common bean with another sole maize were laid out in a factorial arrangement in a randomized complete block design (RCBD) with three replications. The results revealed that the main effect of spatial arrangements highly significantly (*P* < 0.001) affected soil and leaf temperature. Soil moisture content was improved under intercropped plots compared with sole cropping. The intensity of light and qualities, such as red, far-red, and photosynthetically active radiations (*μ*mol m^−2^ s^−1^) and ultraviolet rays (UV)-A, UV-B (W m^−2^), were reduced under intercropping as compared to the sole. Interaction effects of variety, spatial arrangements, and inoculation significantly (*P* < 0.01) affected plant height and leaf area index. Inoculated sole Nassir outperformed for plant height and leaf area index. Inoculated sole Hawassa Dume variety performed best for nodule number plant^−1^, nodule dry weight plant^−1^, pods number plant^−1^, 100 seed weight, grain yield, and above-ground biomass yield. The highest grain yield (2.8 t ha^−1^) was recorded from inoculated sole Hawassa Dume. However, considering the equivalent ratio (LER), intercropping with one maize row to two haricot bean rows spatial arrangements was productive by 62% more than sole cropping (total land equivalent ratio of 1.62%).

## 1. Introduction

Land scarcity is one of the constraints facing smallholder farmers, especially in developing countries of Asia and Africa [1]. In southern Ethiopia, about 30% of farmers have an average land holding of 0.5 to 1 ha and a further 40% have 0.1 to 0.5 ha [2]. To compensate for the land fixed asset, farmers from these fragments of land benefited when they practice intercropping system. Yield is taken as a primary consideration in the assessment of the potential intercropping practices [3]. Farmers will have an advantage from intercropping because of higher yield and greater biological and economic stability, efficient land use, reduced loss of crop due to disease or pest, reduced soil erosion, maximum use of soil moisture, plant nutrients, reduced risk of crop failure, and less weed infestation. Moreover, land degradation has become a global environmental threat and farmers need to adopt sustainable land management and conservation strategies like intercropping [4]. Common bean (*Phaseolus vulgaris* L.) is one of the most important food legume crops for direct consumption in the world [5]. The crop is distributed and grown in different parts of Ethiopia depending on climatic and socio-economic factors [6].

Microclimate including temperature, relative humidity (RH), and light intensity in farmland are important factors in the growth and production of crops. The microclimate of the crop varies from top to bottom of the canopy [7]. Intercropping provided heavy shading on the common bean as the height of the maize is high. Light shading influenced the photosynthetic process. Farrel and Altieri [8] elaborated that as a result of intercropping, microclimate within the canopy can moderate temperature extremes and lower temperatures with reduced air movement leading to decreased evaporation rates and increased relative humidity, which is important in avoiding desiccation and providing favorable growth conditions. Inoculation with effective *Rhizobium* strains substantially increases the nitrogen-fixing potential and yields of legumes, including common beans. However, farmers have a wrong notion that the common bean, being a legume crop, does not need any fertilizer and usually grows on marginal land without applying any fertilizer. This seems to be an important reason for its low seed yield in Ethiopia. This constraint could be alleviated through seed and/or soil inoculation with the proper *Rhizobium* bacteria before or at planting to facilitate N-fixation [9]. Therefore, to increase the productivity of the farmers, it is crucial to increase awareness of farmers towards the utilization of improved agronomic practices that increase their productivity and accelerate food security through proper implementation.

Intercropping has a significant effect on microclimate and resource use efficiency [10]. The previous research work on common beans intercropped with maize has been concentrated on yield and fertilization rate. However, shading may influence the quality of legume crops. The microclimate change induced by another crop species may influence the growth and yield of the physiology of the neighboring crops. The reduction in available light under the shade due to taller component crops increases heights and favors lodging of shorter crops and may impede their growth and yield [11]. Maize grows fast and produces a high leaf area index that provides shade over the soil during the first three months of growth. Mixed crops also deplete soil moisture and nutrient levels because of higher water and nutrient use caused by the rapid development of leaf and root density [12]. The reduction in air temperature under maize and sorghum shades was reported to favor the growth of potatoes and groundnut, particularly when the ambient temperature in a pure potato crop was above optimum [13]. Although maize and common bean are the major crops grown in intercropping systems, there is a limited report on the above- and below-ground resource utilization during intercropping of each crop. The effect of intercropping of maize-common bean on agronomic and yield advantages is well-studied but there is a lack of study on its impact on the microenvironment of the campaign crops. The objectives of this study were first to evaluate the impact of maize, common bean intercropping on microclimate, and to determine the effects of common bean, maize intercropping on the growth, nodulation, and yield of common bean varieties under *Rhizobium* inoculation, and second to evaluate the economic advantage of the intercropping over sole cropping system.

## 2. Materials and Methods

### 2.1. Description of the Experimental Site

The experiment was conducted in the 2020 main cropping season under supplemental irrigation at the experimental field of Hawassa University, Hawassa, Ethiopia. The site is located 270 km south away from the capital city Addis Ababa. Geographically the area lies at 7° 03ʹ 53.8ʺ N and 38° 28ʹ 59.2ʺ E with a mean altitude of 1694 meters above sea level. [14]. The soil of the experimental site was tropical Andosols [15], well-drained sandy clay loam in textural classes with a pH value of 7.2. According to 11 years of observation, the annual total rainfall was about 1274.8 mm with mean minimum and maximum temperatures of 13.6°C and 27.5°C, respectively. The weather conditions are presented (Table 1).

### 2.2. Experimental Materials

The planting materials, common bean varieties (Nassir, Hawassa Dume), and maize hybrid variety (BH-546) were obtained from Hawassa Agricultural Research Center. *Rhizobium* strain (HB-429) was obtained from Menagesha Biotechnology PLC, Addis Ababa, Ethiopia.

### 2.3. Experimental Soil Sampling and Analysis

Before planting, soil samples were taken randomly from the experimental field at 0–20 cm depth using an augur. The samples were mixed well in a plastic bag and sieved, and one composite representative sample was taken for analysis of the physical and chemical properties (pH, total N, available P, and OC) of the soil. The composite soil sample was sent to Debrezeit Horticoop Ethiopia (Horticulture) soil and water analysis laboratory and analysis were performed following the standard procedure for each parameter. The soil texture analysis was performed by the Bouyoucos hydrometer method [16]. The soil analysis result is presented in Table 2.

### 2.4. Treatments and Experimental Design

The experiment consists of two common bean varieties, two inoculation levels (*Rhizobium* strain uninoculated), and three spatial arrangements with sole maize that makes the total treatments thirteen (2 varieties x 2 inoculation x 3 spatial intercropping plus one sole maize). The two common bean varieties (Hawassa Dume and Nassir), two levels of Rhizobium inoculation (I1 = inoculated (HB-429), I2 = uninoculated), and the spatial arrangements of the component crops (sole common bean, maize 1 : 1 common bean, maize 1 : 2 common bean and sole maize plots) were factorially arranged in randomized block design with three replications.

### 2.5. Experimental Design

The space between plots and between blocks was 80 cm and 1 m, respectively. Hybrid maize (BH-546) was planted with 80 × 30 cm inter- and intrarow spacing, respectively, in a plot consisting of four rows of maize. The size of the experimental plot was 3.2 m × 2.10 m (6.72 m^2^), with net and total experimental areas of 262.08 m^2^ and 437.3 m^2^, respectively. Seeds of common bean varieties (Nassir and Hawassa Dume) for *Rhizobium* inoculation treatment were inoculated with HB-429 peat-based carrier as per the recommended rate (10 g inoculant (with charcoal as carrier) per kilogram of seed).

The charcoal base *Rhizobium* inoculum was mixed thoroughly with seeds with a sticker for proper coating. Then, the coated seeds were dried under shade for approximately 25 minutes and then seeded immediately. The detailed procedure is summarized in [17]. Each planting hole received two seeds, which were later thinned into one plant. Uninoculated common bean seeds were planted in their respective plots first and then the inoculated seeds were planted to avoid contamination. Ridges were made to prevent the movement of bacteria through runoff between plots and blocks. Sole common bean planting was 40 × 10 cm inter- and intra row spacing, respectively.

### 2.6. Data Collection and Measurements

#### 2.6.1. Microclimate Data

Soil temperature was measured on each plot using glass thermometer with a red special filling enclosed type, three times per day (at 8: 00 am, 11: 00 am, and 15: 00 (3: 00 pm) hours) for three days total at 5 -day interval during the experimental period (at the midflowering stage). Soil moisture content: during the vegetative growing period, 250 g soil sample was collected per plot and weighed before and after oven drying at 100°C until a constant weight was obtained. Soil moisture content (SMC) was calculated using the weight fraction as [18]

(1) $$\begin{array}{c} SMC\left(\%\right)=\frac{fw-DW}{DW}\times 100\text{,} \end{array}$$

where FW is the fresh weight of soil and DW is the dry (oven-dried) weight of soil.

Leaf temperature was recorded from three randomly selected leaves per plant at central rows using infrared thermometers. The measurement was made three times per day (8:00 am, 11:00 am, and 15:00 (3:00 pm) hours) three days total at 5 -day interval during the midflowering stage of the upper of the common bean leaf. Additionally, light intensity was measured at the seedling stage and vegetative and flowering stages of the common bean crop. The measurement was considered for the intercropped canopy. Ultraviolet (UV)-A and UV-B rays (W m^−2^) and red, far-red, and photosynthetically active radiations (PAR) (*μ*mol m^−2^ s ^−1^) were measured at a one-hour interval from 7:00–17:00 on selected clear sky days using Skye SpectroSense2 and (ultraviolet (UV-B) Skye SpectroSense2 instruments, Llandrindod Wells, UK). For statistical analysis, the mean values of photosynthetically active radiation and ultraviolet A and (UV)-B obtained between 11:00 and 15:00 hours were used.

#### 2.6.2. Growth Parameters

Plant height (cm) was measured at the maturity stage from five randomly selected plants from each plot from the ground level to the apex of the main stem using a tape meter. Leaf area was measured from five randomly selected plants harvested for shoot weight determination from the central rows of each plot at mid flowering stage. The average of the five plants' leaf area was taken as the leaf area of the plant, i.e. leaf number plant^−1^ and leaf area (LA) (cm^2^). It was measured by using a portable leaf area meter (model LI-3000A Li-COR, Lincoln, USA). Then, leaf area indexes (LAI) were calculated by using the following equation [19]:

(2) $$\begin{array}{c} LAI=\frac{total\;green\;leaf\;area\;of\;the\;sampled\;plant}{ground\;area\;occupied\;by\;the\;sampled\;plant}\text{.} \end{array}$$

#### 2.6.3. Nodule Determination

Five randomly selected plant samples were carefully uprooted at the flowering stage and nodules were counted for the total nodule number per plant. Nodules were then dried in an oven for 24 h at 70°C and weighed for determination of nodule dry weight per plant.

#### 2.6.4. Yield-Related Parameters

The number of pods per plant^−1^ was determined from ten plants harvested from three central rows of each plot and the average was taken as the number of pods per plant^−1^. Hundred seed weight (g) was determined by weighing randomly sampled hundred seeds from the total seed threshed for seeds pod^−1^ determination per plot. Counted seeds were weighed using sensitive balance and their average weight was taken as the weight of hundred seeds. Above-ground biomass yield (t ha^−1^) was measured from plants manually harvested from the central rows of each plot. The harvested plants were sun-dried in the open air and the average total biological yield was reported in t ha^−1^. Finally, for grain yield (t ha^−1^), the harvested plants from central rows were threshed and weighed and then converted to t ha^−1^ to determine the grain yield per hectare. Seed yield was adjusted at a 10% moisture level using a digital seed moisture tester and converted to a hectare basis. The adjusted yield was calculated by using the following formula [20]:

(3) $$\begin{array}{c} adj\;usted\;yield=\frac{100-actual\;moisture}{100-standa\;rd\;moisture}\;x\;obtained\;yield\text{.} \end{array}$$

The land equivalent ratio (LER) was used to evaluate the productivity of intercrops compared with mono-crops. It was calculated according to Mead and Willey [21].

(4) $$\begin{array}{c} LER=\frac{Yab}{Yaa}+\frac{Yba}{Ybb}\text{,} \end{array}$$

where Yab = yield per unit area of crop *a* in the intercrop, Yaa = yield per unit area of crop a in the sole crop, Yba = yield per unit area of crop b in the intercrop, Ybb = yield per unit area of crop b in sole crop, *a* = maize, and *b* = common bean.

### 2.7. Statistical Analysis

All data collected were subjected to analysis of variance (ANOVA) appropriate to factorial experiment in an RCBD by statistical analysis system using the General Linear Model SAS version 9.0 [22]. Treatment means were compared using the least significant difference (LSD) at a 5% level of significance.

## 3. Results and Discussion

### 3.1. Effect of Intercropping on the Microclimate of the Campaign Crops

Soil temperature, soil moisture content, and leaf temperature were highly significantly (*P* < 0.001) affected by the main effect of spatial arrangement. However, these microclimate parameters were not significantly affected by the variety, inoculant, and all interaction effects (Table 3).

### 3.2. Soil Temperature

The result indicated that the highest soil temperature (25.40^o^C) was recorded on the sole common bean plot. The lowest soil temperature was recorded in both (spatially arranged) intercropping treatments (Table 4). Increased soil temperature in sole cropping might be due to unshading plots resulting in the highest light intensity. Soil temperature had a strong positive association (*r* = 0.79^*∗∗∗*^) with grain yield. A similar result also reported that the optimal soil temperature for plant growth and uptake of mineral nutrients is 25°C [23]. The lower soil temperature under intercropping treatments could be due to shade from the maize as shaded soils dry out and remain cool than unshaded soil. Canopy microclimate such as light, temperature, or relative humidity is of great significance for crop growth and development, which is influenced greatly by plant densities, suggesting that an increase in planting density significantly reduced both the intensity of light and temperature but increased air relative humidity [24].

### 3.3. Soil Moisture Content

Treatments in a spatial arrangement in the intercropped system resulted in higher soil moisture content as compared to sole cropping (Table 4). The intercropped plots increase soil moisture content by 36.80% compared to the sole plots. Intercropped treatments minimized moisture loss by the shade with the canopy of the maize plants. Thus conserving soil moisture in a dry environment using intercropping may be very useful in improving common bean growth and development. Similarly, Woomer et al. [25] found that under dry conditions, intercropping could have been advantageous for common beans because of the shade provided by maize. Soil moisture content has a strong and highly significant negative correlation with yield and yield components of haricot beans in the intercropping system (Table 5). Unshaded sole plots have a higher yield than intercropped plots due to the absence of competition. Intercropped plots have higher moisture content due to the shading effect but lower yield due to competition.

### 3.4. Leaf Temperature

Higher leaf temperatures (22.30°C, 28.06°C, and 30.95°C) were recorded on sole cropping at different times per day (at 8: 00 am, 11: 00 am, and 15: 00 (3:00 PM hours), respectively whereas the lower leaf temperature was recorded on both double and single-row intercropping treatments (Table 4). An average daily temperature of 26.42^o^C was recorded on the leaves of sole crop plots. Leaf temperature had a strong positive association with a number of pods per plant^−1^(*r* = 0.89^*∗∗∗*^) and grain yield (*r* = 0.84)^*∗∗∗*^(Table 5). This result indicated that sole cropping can optimize the leaf temperature to around 25^o^C suitable for the common bean as described by Singh [26]. Reduced leaf temperature under intercropping might be due to decreased light intensity by the canopy of maize. In addition, the transpiration loss of water from the maize leaf reduced the leaf temperature. Kumar and Tieszen [27]; confirmed the leaf temperature effect in studies where plants experienced a decrease in net CO_2_ assimilation due to a reduction in stomatal conductance for temperatures in the range of 25 to 35°C.

### 3.5. Light Intensity

**Figure 1:**
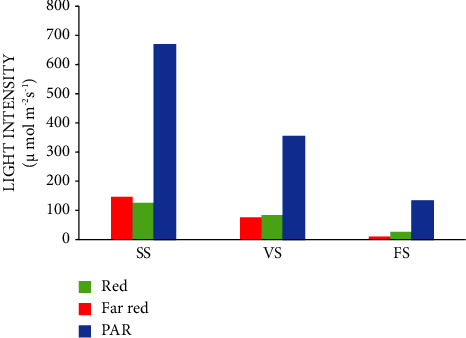
Red, far-red, and photosynthetically active radiations (A) at seedling, vegetative, and flowering stage under the intercropping canopy during the growth period at Hawassa. Where, SS = seedlingstage, VS = vegetativestage, FS = floweringstage.

**Figure 2:**
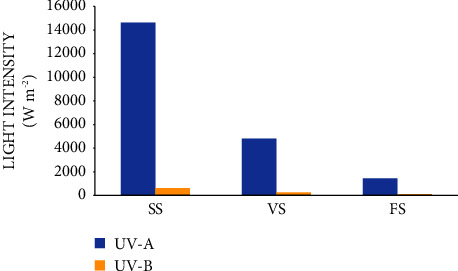
Ultraviolet (UV)-A and UV-B (B) at seedling, vegetative, and flowering stage under the intercropping canopy during the growth period at Hawassa. Where, SS = seedlingstage, VS = vegetativestage, FS = floweringstage.

It was observed that higher light intensity was recorded at the seedling stage of common beans in the experimental field. The results in Figures 1 and 2 indicate that higher levels of red, far-red, and photosynthetically active radiations (PAR) (*μ*mol m ^−2^ s ^−1^), and ultraviolet (UV)-A, UV-B (W m^−2^ s^−1^) rays were recorded at the seedling stage than a vegetative and flowering stage of common bean intercropped treatments. These variations might be due to the light transport with slow as canopy size increased within the vegetative and flowering stages. Light intensity variation within the canopy is affected by leaf shading by other leaves, which varies rapidly [28] and depends on a host of environmental, physiological, and morphological factors. Similar studies reported that the negative effect of shading on soybean growth showed close planting of maize caused severe shading and absorbed most of the light under the maize-soybean relay strip intercropping system [29].

### 3.6. Growth and Nodulation Parameters

#### 3.6.1. Plant Height

The interaction effects of variety, spatial arrangement, and inoculation significantly (*P* < 0.05) affected plant height, leaf area index, nodule number per plant, and nodule dry weight per plant of common bean (Table 6).

#### 3.6.2. Plant Height

The tallest plant (76.4 cm) was recorded in the Nassir variety under sole cropping with inoculated treatment. Plant height was shorter under intercropping conditions of both varieties particularly when they were not inoculated with the *Rhizobium* strain. Among the intercropping interactions, the Nassir variety under inoculated treatment resulted in taller plant height than the other interactions (Table 7). The higher plant height of the Nassir variety may be due to the semi indeterminate growth habit of the variety coupled with improved N nutrition due to inoculation and sufficient light availability due to the absence of shading in the sole cropping. Similarly, Yamanaka et al. [30] reported that there was a significant increase in plant height following *Rhizobia* inoculation. This finding was in agreement with Shahzad et al. [31] who reported plant height is mainly controlled by the genetic makeup of a genotype and it can also be affected by environmental factors. This result is also in agreement with Getahun and Abady [32] who recorded higher plant height from sole cropping in common bean-maize intercropping. The results coincide with the findings of Abbasi et al. [33], who concluded that the treatments of *Rhizobium* inoculation increased soybean plant height by up to 12%. The shorter plants in intercropped and uninoculated treatments might be due to the microclimate effect of maize canopy and reduced light intensity which may result in lower light intensity and physiological process. This result was supported by Niinemets [34] who indicated that a lower plant growth rate, shortage of ATP, and energy supply by photosynthesis is due to the shorter plants' height under microclimate effects.

#### 3.6.3. Leaf Area Index

The highest leaf area index (LAI) (2.65) was recorded from the inoculated sole plot of the Nassir variety, whereas the lowest LAI was recorded in uninoculated and M1 : 1CB intercropped treatments of both varieties (Table 7). The highest LAI of the Nassir variety on a sole plot with *Rhizobium* inoculation might be associated with the various growth habits, the absence of shading effect, and better N supply with *Rhizobium* inoculation gave higher leaf areas than those without inoculation and intercropping. Similarly, Kassahun [35] reported that a sole common bean had a significantly higher leaf area and leaf area index than an intercropped common bean. The increase in LAI in response to R*hizobium* inoculation might be attributed to the availability of N that led to high LAI through facilitated vegetative growth and more expansion of leaves. A similar finding by Majid et al. [36] indicated that inoculation with effective *Rhizobium* strain (HB-429) brought a significant effect on the leaf area index of legumes.

#### 3.6.4. Number of Nodules Plant^−1^

The *Rhizobium* inoculation of the Hawassa Dume variety on the sole plot resulted in the highest average number of nodules plant^−1^ (51.9). When not inoculated with *Rhizobium*, both varieties resulted in the lowest number of nodule plant^−1^ (Table 7). The higher nodule number from inoculated Hawassa Dume variety and sole cropping can be explained by the higher infection rate and compatibility the *Rhizobium* inoculant has with the variety. The increased number of nodules in sole cropping might be due to the reduced competition for resources from the maize crop.

#### 3.6.5. Nodule dry Weight Plant^−1^

The highest nodule dry weight (0.64 g plant^−1^) was recorded on the Hawassa Dume variety planted on sole cropping with inoculated treatment. The lowest nodule dry weight was recorded by both varieties in the intercropping treatments regardless of the inoculation (Table 7). The increment in nodule dry weight of the Hawassa Dume variety under sole cropping with inoculation may be due to the higher infection and compatibility between the variety and the inoculant, and better light use than uninoculated and sole cropping. Effective light use might be due to better soil nutrition for more nodule formation. Nyoki and Ndakidemi [37] reported a similar promoting effect of seed inoculation on the dry weight of nodules plant^−1^. Dereje [38] also reported similar effects of seed inoculation on nodule dry weight. This result is also in agreement with the work of Yoseph et al. [39], who reported marked differences among the cowpea varieties on nodule dry weight plant.

#### 3.6.6. Treatments Effect on Yield and Yield Components

The analysis of variance indicated that the three-way interaction of variety, spatial arrangement, and inoculation significantly affected the number of pods per plant and grain wait of common bean (Table 8). On the other hand, hundred seed weight and above-ground biomass were affected by the two-way interaction of spatial arrangement and Rhizobium inoculation but not the three-way interaction. However, hundred seed weight was not significantly affected by the spatial arrangement and the two-way interaction of variety and spatial arrangement, variety, and inoculation, and the three-way interaction of variety, spatial arrangement, and inoculation (Table 8).

#### 3.6.7. Number of Pods per Plant

The highest number of pods per plant was recorded from inoculated under sole cropping on the Hawassa Dume variety, whereas the lowest number of pods per plant^−1^ was recorded in uninoculated and intercropped treatments of both varieties (Table 9). The improvement in nodule number for the inoculation treatment can be associated with enhanced N nutrition due to N_2_ fixation. This is because an improved N supply improves light use efficiency and reduces abortion and the abscission of flowers and pods [40]. The difference in pod number per plant among the varieties can be related to the yielding capacity of the varieties. Similarly, Argaw [41] also reported that the number of pods per plant increased due to *Bradyrhizobium* inoculation in soybean. The current result is in agreement with the work of Dereje [38] who reported an increased number of pods per plant^−1^ with inoculation in green gram and soybean. The lower number of pods per plant in both varieties which were inoculated and intercropped might be due to the microclimate effects of maize as the main crop which caused a reduction in photosynthetically active radiation (PAR). A similar finding by Carruthers et al. [42] related this reduction of photosynthesis due to the shading of associated crops to a level that the legume plants compensated by decreasing the amount of assimilate allocation to reproductive growth or grain production. This result is in line with the findings by Ndakidemi and Dakora [43], who reported a reduction in cowpea number of pods per plant under intercropping compared to sole cropping.

#### 3.6.8. Grain Yield

The highest grain yield (2.78 t ha^−1^) was produced from the Hawassa Dume variety with *Rhizobium* inoculation on sole plots. While one-to-one (M1 : 1CB) intercropping resulted in similar grain yield across all varieties and inoculation treatments. This indicates that lower grain yield was recorded when both varieties were arranged with an M1 : 1CB ratio regardless of the inoculation (Table 9). Higher grain yield for inoculated sole cropping plot of the Hawassa Dume variety might be related to the differences in the genetic makeup of the variety, no competition with maize to capture environmental resources (water, light intensity, and soil nutrients) with good efficiency of N_2_ fixation. This treatment also demonstrated higher performance for yield contributing parameters including the number of pods per plant and seeds per pod. Similar to this result Haruna and Usman [44] observed a significant variation in grain yield of some improved varieties of cowpea and attributed it to the genetic makeup of the varieties examined. The results coincide with the findings of Tarekegn and Serawit [45], who concluded improved seed yield due to *Rhizobium* inoculation in haricot bean varieties. Lower grain yield from M1 : 1CB arrangement might be associated with the microclimate effects of maize canopy and higher competition due to the extensive root system of maize. A similar result was found by Walelign [46], who indicated 80% common bean varieties yield reduction under maize intercropping. Previously, it has been found that severe shading conditions significantly decreased the soybean yield and yield components [47].

#### 3.6.9. Hundred Seed Weight

The cropping system coupled with inoculation affected the hundred seed weight. The highest hundred seed weight (35.56 g) was recorded on sole cropping with the inoculated plot (Table 10). This might be due to higher light intensity and soil fertility as a result of *Rhizobia* inoculation. This result was supported by earlier studies by Ali et al. [48] where inoculation brought a significant effect on the seed weight, of chickpeas. A similar result was also reported by Kazemi et al. [49] who reported that soybean seed inoculation with *Bradrhizobia* significantly increased seed weight.

The lowest hundred seed weight was recorded on sole cropping with uninoculated (Table 10). The reduction in 100 seed weight on the sole crop with uninoculated might be due to the lack of N for photosynthesis that limited seed size. This result is in line with the finding of Wright [50] who reported that a higher hundred seed weight of soybean was recorded under intercropping than sole cropping. This variation might be the effect of shading and reduced sunlight in intercropping than sole cropping.

#### 3.6.10. Above-Ground Biomass

The highest above-ground biomass (12.30 ton ha^−1^) was recorded from inoculated sole cropping. One-to-one intercropping arrangement (M1-1CB) with and without inoculation resulted in the lowest aboveground biomass (Table 10). The above-ground biomass production in this experiment was highly responsive to sole cropping and *Rhizobium* inoculation (HB-429). This might be due to the important role of sunlight in an open area for better-intercepted light and the *Rhizobium* inoculant (HB-429) add N which might result in higher biological yield. This finding was in agreement with Legesse et al. [51] who reported that the highest biomass yield (kg/ha) was obtained from sole fava bean. Similarly, Abbasi et al. [33] also reported that above-ground total biomass yield of soybean was increased by up to 75% by the inoculation of different strains of rhizobia as compared to uninoculated. Other researchers found that biomass accumulation is directly associated with the availability of light intensity and reductions in light intensity decreased biomass production.

The reduction of above-ground biomass under the intercropped might be due to the effect of shading of main crops resulting in lower aboveground biomass because of reduced plant growth. This result conforms with the finding reported by Chui [52] where intercropping reduced soybean biological yield by 87% when compared with sole cropping, principally because of reduced plant growth and photosynthetic assimilation [53]. In addition, Getachew et al. [54] under maize/legume intercropping concluded that aboveground dry biomass yield was significantly reduced by 74% in the intercropping as compared to the sole cropping system.

#### 3.6.11. Biological Productivity of Intercropping

The analysis showed that only the main effect of spatial arrangement significantly(*P* < 0.001) affected the partial land equivalent ratio (LER) of common bean and the total land equivalent ratio (Table 11). However, the partial land equivalent ratio of maize was not affected by any of the factors and their interactions.

#### 3.6.12. Partial Land Equivalent Ratio (LER) of Common Bean

The highest partial LER of common bean (0.66) was found at double row common bean with one row of maize (M1 : 2CB) (Table 12). The overall partial LER of common bean increased the population density increased in all maize-common bean combinations probably due to the efficient utilization of resources. In line with this result, Niringiye et al. [55] reported that intercropping of maize with a different population density of common bean resulted in more yield and economic advantage than sole cropping of the component crops. Similarly, Tilahun [56] reported the effect of plant density and arrangement of component crops on the productivity of the maize/faba bean intercropping system.

#### 3.6.13. Partial Land Equivalent Ratio of Maize

The partial land equivalent ratio (PLER) of maize was not significantly affected by variety, inoculation, or spatial arrangement (Table 12). A mean partial LER of 0.95 was obtained for maize (Table 12). The high partial LER value recorded for maize in all treatments indicated the presence of greater competitive capacity of maize against common bean. More importantly, maize had relatively larger upper canopy structures and the roots of maize grow into larger areas compared to the common bean. The maize component derives its competitive ability from its more resource use efficient C_4_ pathway than the common bean C_3_ photosynthetic pathway [57].

#### 3.6.14. Total Land Equivalent Ratio

Total land productivity is the functions of both crops combined on the same land. The highest total land equivalent ratio (1.62) was found at double row common bean with one row of maize (M1 : 2CB) (Table 12). These results indicated that intercropping system gave 62% yield more land use advantage and profit than needed by producing the two crops together than planting sole cropping of each. Similar to the current result, Tolera et al. [58] reported more yield and higher land use efficiency by intercropping of maize with climbing beans. A land equivalent ratio greater than unity has been reported in maize/faba bean intercropping [56]. However, it is observed that the optimum row arrangement might to achieve at certain points these results of the LER intercropping pattern compared to sole crops might be a better use of land, water, and nutrient. This result was in agreement with the report of Lulie et al. [59] where the LER of maize/common bean ranged from 1.29–1.69 in Ethiopia.

#### 3.6.15. Association of Microclimate Parameters with Major Yield and Yield Components of Haricot Bean

Soil temperature was strongly, positively, and highly significantly correlated with the number of pods above-ground biomass and grain yield of common beans. A similar trend was also observed in the association between leaf temperature and the yield component parameters such as the number of pods above-ground biomass and grain yield, whereas soil moisture content was strongly, negatively, and highly significantly correlated with the major yield components and yield parameters (Table 5). These relationships contradicted the usual trend taking into consideration the importance of soil moisture for plant growth and yield. However, the trail is an intercropping trail in that yield and yield components are obtained from sole vs intercropping plots. By virtue of reality, sole cropping resulted in higher productivity due to the absence of completion for environmental resources such as light, nutrients, space, and other microclimate variables (Table 9). However, the moisture content is higher under intercropped crops due to the shading effect of the main crop (maize). This resulted in a negative correlation of soil moisture content with major yield and yield components. Other studies also show increased soil moisture content but decreased yield and yield components under the intercropping than the sole cropping of the campaign crop [60]. Additionally, as the research was conducted under supplemental irrigation, the influence of soil moisture conservation had little effect on the yield of the crop under intercropping.

## 4. Conclusion

In this study, inoculated sole common beans resulted in better nodule formation, growth, and economic yield compared with the intercropping systems. A strong and positive significant correlation was observed among the major yield parameters and microclimate variables. The microclimate was influenced by cropping systems as intercropping reduced light interception and photosynthetically active radiation (PAR). The highest grain yield (2.78 t ha^−1^) was recorded from HB-429 inoculated sole Hawassa Dume variety. However, sole cropping of common beans was not economically visible considering the land equivalent ratio (LER). Therefore, maize, common bean intercropping with one maize to two common bean row arrangements, can be recommended for higher productivity.

## Figures and Tables

**Table 1:** Ten years (2010–2019) average and the year 2020 cropping season weather data at Hawassa [14].

Month	Ten years average data			2020 data		
	Min. temp. (ºc)	Max. temp. (ºc)	Rainfall (mm)	Min. temp. (ºc)	Max. temp. (ºc)	Rainfall (mm)
January	12.3	29.2	13.2	13.1	28.6	24.3
February	13.3	30.5	26.5	13.7	30.1	61.2
March	14.5	30.6	69.4	15.6	29.8	118.2
April	14.9	29.3	130.0	15.3	29.0	215.2
May	15.2	27.8	156.5	15.5	28.0	199.4
June	15.5	26.2	106.3	15.3	26.1	104.0
July	14.8	25.2	130.8	15.2	24.3	104.2
August	14.7	25.3	121.1	15.4	24.7	130.0
September	14.4	26.0	120.8	15.0	26.0	133.7
October	13.8	27.4	77.5	13.9	27.4	169.9
November	12.7	28.1	45.9	11.4	28.1	17.3
December	11.4	28.2	6.4	10.4	28.3	0.0

**Table 2:** Physical and some chemical characteristics of experimental soils.

Physical properties				Chemical properties			
Soil texture (%)			Texture class	pH (H_2_O)	Total *N*	Av. P (%) (ppm)	OC (%)
Silt	Clay	Sand					
28	31	41	Clay loam	7.1	0.12	58	1.40

**Table 3:** Mean squares from analysis of variance (ANOVA) for soil moisture content (SMC %), soil temperature (ST ^o^C), and leaf temperature (LT ^o^C) common bean.

Sources of variations	Degree of freedom	Mean squares		
		Soil moisture content (%)	Soil temperature (°C)	Leaf temperature (°C)
Replication	2	7.147	1.1506	0.119
Variety (V)	1	7.101 ns	1.0180 ns	0.474 ns
Spatial arrangement (SA)	2	275.319^*∗∗∗*^	80.0880^*∗∗∗*^	101.844^*∗∗∗*^
Rhizobium inoculation (RI)	1	0.365 ns	0.0093 ns	0.0331 ns
V*∗*SA	2	0.253 ns	0.8453 ns	0.116 ns
V*∗*RI	1	1.849 ns	0.7210 ns	0.059 ns
Sa*∗*RI	2	4.320 ns	0.0115 ns	0.230 ns
V*∗*SA*∗*RI	2	1.494 ns	0.2356 ns	0.048 ns
Error	22	2.804	0.3100	0.522

*∗*(*P* < 0.05), ^*∗∗*^(*P* < 0.01), ^*∗∗∗*^ (*P* < 0.001), and ns (*P* > 0.05) indicate significance, highly significant and very highly significant, and nonsignificant variations, respectively.

**Table 4:** Main effects of spatial arrangement on soil temperature (^o^C), soil moisture content, and leaf temperature (^o^C) at different time intervals of common bean.

Treatments	Parameters						
	Soil temperature (^o^C)			Soil moisture content (%)	Leaf temperature (^o^C)		
Variety	8:00 am	11:00 am	3:00 pm	28.61a	8:00 am	11:00 am	3:00 pm
Nassir	18.06a	9.79a	22.57a		20.72a	25.11a	28.69a
Hawassa Dume	17.82a	19.55a	22.23a	29.25a	20.95a	25.44a	28.46a
Spatial arrangement							
Sole	18.96a	20.80a	25.40a	23.53b	22.30a	28.06a	31.95a
M1:1CB	17.32b	19.13b	20.96b	31.19a	20.05b	23.90b	26.76b
M1:2CB	17.54b	19.06b	20.84b	31.22a	20.17b	23.86b	27.02b
Inoculation							
Inoculated	18.01a	19.88a	22.38a	29.16a	20.85a	25.60a	28.61a
Uninoculated	17.98a	19.66a	22.42a	28.95a	20.82a	24.95a	28.55a
LSD (0.05)	0.81	0.78	1.03	2.66	0.89	2.08	1.18
CV %	2.64	2.34	2.49	5.76	1.69	4.38	2.53

Means followed by the same letter in each treatment and parameter are not significantly different at *P* < 0.05 level of significance.

**Table 5:** Linear correlation among microclimate, yield, and yield components of common bean.

	LT	SMC	ST	NPPP	HSW	GY	AGB
LT	—						
SMC	−0.88^*∗∗∗*^	—					
ST	0.83^*∗∗∗*^	−0.79^*∗∗∗*^	—				
NPPP	0.89^*∗∗∗*^	−0.80^*∗∗∗*^	0.79^*∗∗∗*^ -	—			
HSW	0.02ns	−0.002ns	0.06ns	0.36*∗*	—		
GY	0.84^*∗∗∗*^	−0.76^*∗∗∗*^	0.79^*∗∗∗*^	0.97^*∗∗∗*^	0.37*∗*	—	
AGB	0.79^*∗∗∗*^	−0.72^*∗∗∗*^	0.74^*∗∗∗*^	0.91^*∗∗∗*^	0.35*∗*	0.96*∗∗*	—

where ns, *∗*, ^*∗∗*^, and ^*∗∗∗*^ = correlation is significant at *P* > 0.05, *P* < 0.05, *P* < 0.01, and *P* < 0.001 levels of probability, respectively. LT = leaf temperature (^o^C), SMC = soil moisture content, ST = soil temperature (^o^C), NPPP = number of pods per plant^−1^, HSW = hundred seed weight, GY = grain yield, and AGB = above-ground biomass.

**Table 6:** Mean squares from analysis of variance (ANOVA) for plant height, leaf areas index, the number of nodules per plant^−1^, and nodule dry weight per plant^−1^ of common bean.

Sources of Variations	Degree of freedom	Mean squares			
		Plant height (cm)	Leaf area index	Nodule number	Nodule dry weight (g)
Replication	2	11.54	0.0210	21.570	0.0005
Variety (V)	1	628.34^*∗∗∗*^	0.1456^*∗∗∗*^	91.075*∗*	0.0140^*∗∗*^
Spatial arrangement (SA)	2	2545.6^*∗∗∗*^	3.7427^*∗∗*^	784.6^*∗∗∗*^	0.1750^*∗∗∗*^
Rhizobium inoculation (RI)	1	631.68^*∗∗∗*^	0.3864^*∗∗∗*^	589.35^*∗∗*^	0.1122^*∗∗∗*^
V*∗*SA	2	33.95^*∗∗*^	0.03441^*∗∗∗*^	7.841 ns	0.00148 ns
V*∗*RI	1	13.20 ns	0.00047 ns	138.376^*∗∗*^	0.01174*∗*
SA*∗*RI	2	111.34^*∗∗∗*^	0.09954^*∗∗∗*^	148.441^*∗∗*^	0.05063^*∗∗∗*^
V*∗*SA*∗*RI	2	30.97*∗*	0.01808^*∗∗*^	83.71^*∗*^	0.00468^*∗*^
Error	22	7.19	0.00348	15.857	0.00168

*∗*(*P* < 0.05), ^*∗∗*^(*P* < 0.01),^*∗∗∗*^ (*P* < 0.001), and ns (*P* > 0.05) indicate significant, highly significant and very highly significant, nonsignificant variations respectively.

**Table 7:** Interaction effects of variety, inoculation, and spatial arrangement on growth and nodules of common bean.

Treatment variety	Inoculation	Spatial arrangement	Parameters			
			Plant height (cm)	Leaf area index	Nodules N	Nodule dry weight (g)
Hawassa Dume	Inoculated	Sole	64.4b	2.36b	51.90a	0.64a
		M1:1CB	35.8df	1.32g	29.23c	0.26cd
		M 1:2CB	36.0df	1.40ef	27.70cd	0.26cd
	Uninoculated	Sole	49.1c	1.99d	33.40c	0.32c
		M1:1CB	34.5ef	1.25g	23.90f	0.21d
		M1:2CB	31.1f	1.38ef	24.20f	0.21d

Nassir	Inoculated	Sole	76.4a	2.65a	43.50b	0.49b
		M1:1CB	48.3c	1.42e	23.90ef	0.22d
		M1:2CB	40.3d	1.41ef	25df	0.22d
	Uninoculated	Sole	61.2b	2.20c	30.80cd	0.31c
		M1:1CB	37.3df	1.24g	24.60ef	0.20d
		M1:2CB	37.7df	1.41ef	24.40ef	0.20d

LSD(0.05)			4.63	0.11	6.81	0.06
CV %			5.82	3.55	13.12	13.92

Means followed by the same letter in each column are not significantly different at *P* < 0.05 level of significance.

**Table 8:** Mean squares from analysis of variance (ANOVA) for number of pods per plant^−1^ (NPPP), hhndred seed weight (100SW), grain yield (GY), and above-ground biomass (AGB) common bean.

Sources of variations	Degree of Freedom	Mean squares			
		Number of pods per plant	100 seed weight	Grain yield	Above-ground biomass
Replication	2	0.203	1.810	0.00310	0.3217
Variety (V)	1	1.822 ns	21.007*∗*	0.06588^*∗∗*^	0.7168 ns
Spatial arrangement (SA)	2	413.932^*∗∗∗*^	0.135 ns	2.95187^*∗∗∗*^	34.8601^*∗∗∗*^
Rhizobium inoculation (RI)	1	55.007^*∗∗∗*^	174.680^*∗∗∗*^	0.53778^*∗∗∗*^	6.9344^*∗∗∗*^
V*∗*SA	2	0.907 ns	0.264 ns	0.06092^*∗∗*^	0.7899 ns
V*∗*RI	1	11.674^*∗∗*^	11.674 ns	0.07654^*∗∗*^	0.8040 ns
SA*∗*RI	2	37.730^*∗∗∗*^	76.419^*∗∗∗*^	0.23900^*∗∗∗*^	1.5526*∗*
V*∗*SA*∗*RI	2	4.872*∗*	10.527 ns	0.05145^*∗∗*^	0.7659 ns
Error	22	1.236	4.119	0.00737	0.3651

*∗*, (*P* < 0.05), ^*∗∗*^ (*P* < 0.01), ^*∗∗∗*^ (*P* < 0.001), and ns (*P* > 0.05) indicate significant, highly significant and very highly significant, and nonsignificant variations, respectively.

**Table 9:** Interaction effects of variety x inoculation x spatial arrangement on yield component of common bean.

Variety	Treatments inoculation	Spatial arrangement	Parameters	
			No of pods per Plant^−1^	Grain yield (t ha-1)
Hawassa Dume	Inoculated	Sole	24.3a	2.78a
		M1:1CB	9.3d	1.36ef
		M 1:2CB	9.8d	1.55d
	Uninoculated	Sole	15.1c	1.97c
		M1:1CB	8.7d	1.30f
		M1:2CB	8.7d	1.45de

Nassir	Inoculated	Sole	20.7b	2.29b
		M1:1CB	9.3d	1.34ef
		M1:2CB	9.2d	1.54d
	Uninoculated	Sole	16.7c	1.96c
		M1:1CB	8.4d	1.27f
		M1:2CB	9.5d	1.44de

LSD (0.05)			0.14	1.82
CV %			5.23	8.96

Means followed by the same letter in each column are not significantly different at *P* < 0.05 level of significance.

**Table 10:** The interaction effects of spatial arrangement and inoculation on above-ground biomass and hundred seed weight of common bean.

Treatments	Parameters			
Spatial arrangement	Above-ground biomass(t ha^−1^)		100 seed weight (g)	
	Inoculated	Uninoculated	Inoculated	Uninoculated
Sole	12.30a	10.6b	35.56a	25.33c
M1:1CB	8.26de	7.93e	31.20b	29.73b
M1:2CB	9.55c	8.95cd	31.03b	29.51b
LSD 0.05	1.01		3.33	
CV%	6.29		6.68	

Means followed by the same letter (s) in the same parameters are not significantly different at *P* < 0.05 level of significance.

**Table 11:** Mean squares from analysis of variance (ANOVA) for partial and total land equivalent ratio from the common bean-maize intercropping.

Sources of variations	DF	Mean squares		
		Partial common bean	Partial maize	Total land equivalent ration
Replication	2	0.00001	4.174*E* − 05	0.00010
Variety (V)	1	0.00001ns	7.531*E* − 06ns	0.00003ns
Spatial arrangement (SA)	1	0.06153^*∗∗∗*^	4.570*E* − 05ns	0.06493^*∗∗∗*^
Rhizobium inoculation (RI)	1	0.00165ns	9.528*E* − 05ns	0.00254ns
V*∗*SA	1	0.00029ns	8.656*E* − 05ns	0.00069ns
V*∗*RI	1	0.00008ns	3.214*E* − 06ns	0.00011ns
SA*∗*RI	1	0.00134ns	3.337*E* − 05ns	0.00180ns
V*∗*SA*∗*RI	1	0.00001ns	4.802*E* − 06ns	0.00003ns
Error	14	0.00212	4.225*E* − 04	0.00235

^*∗∗∗*^ (*P* < 0.001) and ns (*P* > 0.05) indicate very high significance.

**Table 12:** Partial and total land equivalent ratio (LER) from the common bean-maize intercropping.

Treatments	Partial LER		
	Common bean	Maize	Total LER
Variety			
Hawassa Dume	0.61a	0.95a	1.56a
Nassir	0.61a	0.95a	1.56a
Spatial arrangement			
M1:1CB	0.56b	0.95a	1.51b
M1:2CB	0.66a	0.95a	1.62a
Inoculation			
Inoculated	0.62a	0.95a	1.57a
Uninoculated	0.60a	0.95a	1.55a
LSD ( 0.05)	0.05	0.1	0.05
CV %	7.50	0.68	3.09

Means followed by the same letter in each treatment and parameter are not significantly different at *P* < 0.05 level of significance.

## Data Availability

All data are provided in the article.
